# Supplementary material for: In–silico simultaneous respiratory and circulatory measurement during voluntary breathing, exercise, and mental stress: A computational approach
Source: PLoS Comput Biol. 2024 Dec 17;20(12):e1012645. doi: 10.1371/journal.pcbi.1012645 (PMC11651585; doi:10.1371/journal.pcbi.1012645)
Supplement: Attachment — Submitted filename: Rebuttal_letter_responses_to_reviewers.pdf [file pcbi.1012645.s001.pdf]

Reviewer #1:

Thank you for reviewing this manuscript. Your feedback has significantly improved the quality of our manuscript. Please find our detailed responses and corresponding revisions highlighted in yellow color in the re-submitted documents.

In "Computational approach for predicting respiratory and circulatory system responses to voluntary breathing or exercise" by Iwamoto et al., the authors have expanded upon a model originally developed by Molkov and expanded upon by these authors. This iteration of the model includes voluntary breathing, exercise included changes in physiological values, and an abstraction of psychological stress.

I find the manuscript very interesting, well written, and a contribution to the corpus of computational models focused on control of breathing and the cardiovascular system. The in silico experiments are representative of prior work using human subjects and the authors do a very good job of describing the limitations of their model and the potential reasons for mismatches between the model and actual human subject data. I have only one major concern and that is:

I would like to see other metrics of heart-rate variability (SDNN, sample-entropy, Poicare maps), rather than just the PSD. There is some controversy about whether LF/HF ratios (and overall PSD) clearly represent sympathetic and parasympathetic tone. I realize this requires more work but would provide a foundation for further validation in human subject data. Additionally, in Figure 8, I would like to see the summed PSDs divided to show values for VLF, LF, and HF rather than the totals of all power spectral density for each test condition.

➔ We greatly appreciate you for your comments on the advantage of our model. Other metrics of heart-rate variability (HRV) helps us understand how HRV indices change and influence human physical and mental state during voluntary breathing (VB), short-term exercise (STE), or mental stress (MS) loading. We have added new figs. 6 and 7, representing (a) SDNN, (b) Sample Entropy, (c) Total power of HRV, and (d) LF/HF power ratio and Poincare plots, respectively. We also added new paragraphs to explain how HRV indices change during VB, STE, or MS to lines 192–209 in the Results section. According to HRV indices, deep slow VB at 6 bpm increased the total HRV power, SDNN, and ellipse area of Poincare plot (EAPP), thereby enhancing cognitive performance, variability, and relaxation, whereas MS loading elevated the sample entropy and reduced the total HRV power and EAPP, leading to decreased relaxation. This explains how HRV indices influence human mental state. We have added some sentences to lines 304–315 in the Discussion section.

As a comment, I am not sure that the conglomerate "psychological stress loads" as modeled by the authors are accurate representations of psychological stress but I find their implementation in the model very interesting and think that these parameters can be more nuanced by the inclusion of experimental data. Regardless, the predictions are an intriguing addition to the model and simulated experiments. As the authors mention, inclusion of the amygdala-prefrontal cortex-hippocampus circuit would be worth pursuing in a future version of the model. Obviously, the authors have developed the model in a way that allows modular inclusion of these important physiological interactions as more data is available.

➔ Thank you for your positive comments. We are also not sure that “psychological stress loads” accurately represent psychological stress; however, the current model did not include the amygdala-prefrontal cortex-hippocampus circuit and the input data delivered to the amygdala under psychological stress loads remain unclear. We wish to add the amygdala related circuit to the model in future studies.

Minor concerns include:

1. Abstract. "fragmental" should be "fragmentary".

→ We have omitted this term in the Abstract.

2. Lines 82 to 83. "respiratory center and system" sounds odd and is not commonly used within the field of control of breathing. I would suggest "respiratory rhythm-generation and pattern formation regions"?

→ We have revised the phrase in line 358 as per your suggestion.

3. Line 319. The "vertical vibration of the heartbeat" should be simplified to "maximum heart rate".

→ We have revised the phrase in line 250 as per your suggestion.

4. Why include the u_lm parameter (laryngeal muscles) if they were not modeled?

→ We have omitted the corresponding sentence because the u_lm was set to zero as per your suggestion.

5. Lines 473 to 474. I think the authors mean that they are adapting the rodent respiratory model to humans. The way it is written here, it sounds backwards.

→ We sincerely apologize for the error. We have modified adult rat to adult human in line 438.

6. Line 554. The apostrophe is typeset incorrectly with adjacent spaces.

→ We have revised it in line 536.

7. I was unable to obtain the code from the Zenodo link specified. Please provide a valid link to the model code.

→ We sincerely apologize for this inconvenience. We have provided a valid link to the model code as follows: “The source code and data used to produce the results and analyses presented in this manuscript are available for anonymous peer-review at https://osf.io/k46r8/?view_only=d2fa6f3f571348a1b5c08ce515df594f." in lines 589–592.

Reviewer #2:

Thank you for reviewing our manuscript. Your feedback has significantly improved the quality of our work. Kindly find our detailed responses and corresponding revisions highlighted in green color in the re-submitted documents.

This study provides a computational model of cardiorespiratory control, incorporating central and peripheral neural elements, gas exchange, ventilation, and cardiovascular state variables and parameters. The model is based on previous work on neural circuit models of respiratory and cardiovascular function. The authors focus on modeling cardiorespiratory responses to various types of perturbation, viz., mental stress, voluntary breathing maneuvers, and bouts of exercise.

My concern is the lack of a clear foundational area of biology and medicine this model is advancing. What scientific principles are revealed by the model per se that have not previously been described in the empiric work? Readers might view the scope of this work as quite limited: assembling a high-dimensional model and parameter-fitting to the empiric results. I was unable to find any truly predictive results, i.e., simulated results that reveal new insights and are not already found in the published experimental literature. If such simulated novel insights are presented, the authors should make this case more apparent to the general reader.

➔ Your comments are partially correct. The proposed model was used to reproduce the existing experimental measurements and not used to reveal new insights in the fields of biology and medicine. Therefore, we completely understand your concern. However, we propose a computational approach for *in-silico* simultaneous measurement of multiple physiological values: respiratory states, heart rate, heart rate variability, and blood pressure, that is impossible *in-vivo*, particularly during active states. To t our knowledge, no studies on simultaneous measurement of the multiple physiological values exist *in-silico* and *in-vivo*. Since the physiological values interact with each other, we cannot comprehend the overall responses of the respiratory and circulatory system sustained from external stimulations, which are critical in the fields of biology and medicine. Our proposed model would contribute to revealing unknown mechanisms in respiratory and circulatory systems.

   We have revised the title and sentence in the abstract and lines 63–67 and 70–73 in the

   Introduction section to emphasize our claim and have added some sentences to explain that our proposed model is needed for *in-silico* simultaneous measurements critical in the fields of biology and medicine.

I have additional questions/concerns related to the model as related to cardiorespiratory control

during exercise:

1. The comparisons of simulated and actual responses do have some large differences that are not fully discussed. What aspects of the model account for the deviations. For example, Figures 3 and 4 at the onset there appear to be large differences. The authors have not subjected their model to VVUQ (Verification, Validation and Uncertainty Quantification).

→ We sincerely apologize for insufficient descriptions on the disagreement of tidal volume in Figs. 3 and 4. We used a closed-loop model proposed by Molkov et al. (2014), which includes a robust algorithm to control respiratory autonomously, similarly to living animals and humans. Even if abnormal respiration is generated, the algorithm can promptly return to a stable respiratory state by using mechanical and chemical feedbacks. Thus, after the initial parameters are input to the model, almost 3 cycles of respiration with a time period of approximately 15 s are needed for a stable respiration in the resting state. We included a process until the stable respiration in the original manuscript; however, the process is not a target that we should compare with experimental data. Therefore, we omitted the process from initial to 20 s in Figs. 1–5 of the revised manuscript for valid comparisons between model results and experimental data. We have added some sentences in lines 575–579 in the Materials and methods section.

2. The model lacks important aspects of respiratory and cardiovascular control physiology. For example, short-term and long-term potentiation of active respiration. The role of potassium in exercise hyperpnea. Mechanoreceptor feedback control of cardiorespiratory control during exercise. Entire areas of modeling and discussion of established literature are missing.

→ We think that the relationship between minutes ventilation (MV) and blood oxygen concentration during exercise is the most important aspect concerning respiratory and cardiovascular control physiology. Our model predicted an increase in the blood oxygen pressure and decrease in the blood carbon dioxide during active exercise (Fig. 5), thereby causing exercise hyperpnea. Decrease in the blood carbon dioxide concentration leads to decrease in the blood potassium concentration, thereby causing numbness and more severe hyperpnea. Additionally, increase in the MV with dead space, that is, with larger airway resistance generates short-term and long-term potentiation of active respiration (Babb et al. 2010). Therefore, prediction of MV along with change in the PCO_2 ($PaCO_2$ in the revised manuscript) or PO_2 (PaO_2 in the revised manuscript) during active exercise is critical to understand respiratory and cardiovascular responses during exercise. Moreover, our model includes mechanical and chemical feedback control of cardiorespiratory control during

exercise. Therefore, we do not think that our model lacks important aspects of respiratory and cardiorespiratory control physiology. We have added some sentences to lines 13−17 in the Introduction section and cited new references [15,16] (Miyamoto et al. 2004, Babb et al. 2010).

[15] Miyamoto T, Inagaki M, Takaki H, Kawada T, Yanagiya Y, Suribachi M et al. Integrated characterization of the human chemoreflex system controlling ventilation, using an equilibrium diagram. Eur J Appl Physiol. 2004;93:340-346.

[16] Babb TG, Wood HE, Mitchell GS. Short-and long-term modulation of the exercise ventilatory response. Med Sci Sports Exerc. 2010:42(9):1681-1687.

3. Twenty-second bouts of exercise is too brief to capture the key mechanisms of cardiorespiratory control during exercise. Exercise has several canonical phases that evolve over minutes. The changes in ventilation, acid-base balance, and gas levels should be studied over many minutes if the goal is to understand an integrated systems control of exercise. The short time frames described here may miss important features and is insufficient for validation and error-estimation of the model.

→ We completely agree that 20-second bouts of exercise are too brief to capture the key mechanisms of cardiorespiratory control during exercise. However, our goal was not to understand the integrated system controlling exercise. Our goal was to investigate effects of external stimulations on emotions. In this study, exercise is one of the external stimuli. Since emotion is considered a physiological and/or psychological state during a short-term period, we focused on short-term exercise in this study. We have revised the term "exercise" to "short-term exercise (STE)" in the revised manuscript.

It is not clear whether the authors are familiar or have considered previous work relevant to #2 and #3. Although a bit dated, an example of a comprehensive review of the older literature can be found in Waldrop, T. G., Eldridge, F. L., Iwamoto, G. A., & Mitchell, J. H. (2010). Central neural control of respiration and circulation during exercise. Comprehensive Physiology, 333-380. This review has several results from computer simulations that might be of interest as a point of comparison. For example, the older models do not include detailed ionic and circuit based modeling used in the current manuscript. What specific insights have the current authors gained from their simulation that are possible only by inclusion of this level of neuronal and circuit detail? I was unable to find such lessons in the current manuscript and this could be a missed opportunity for scientific advancement revealed by the computational model.

→ As you have accurately mentioned, the model described in the review paper by Waldrop et

al. (2010) does not include detailed ionic and circuit based modeling but simulates only ventilation and PCO_2 time histories during 10-minutes exercise. However, the model does not predict heart rate and blood pressure because more complex control systems, such as RVLM and AMB, are necessary for understanding appropriate circulatory responses as pointed out by Waldrop et al. (The heart rate and blood pressure shown in the paper are all experimental data**)**. On the other hand, our model can predict heart rate, heart rate variability, and blood pressure in addition to respiratory states, such as breathing rate, tidal volume, ventilation, PO_2, and PCO_2. The model described in the review paper cannot comprehend effects of the circulatory system on the responses during exercise differently from our model. Additionally, as mentioned in the response to your comment #3, our goal was to investigate the effects of external stimulations on emotions. Therefore, detailed ionic and circuit based modeling is necessary to receive various stimulations and connect several neuron groups related to emotional outputs such as the amygdala and locus coeruleus. Thus, our model has scientific advancement for future application for improving physical and/or mental health.

We have added a paragraph to lines 23–38 in the Introduction section and cited new

references [18,19] (Waldrop et al. 2011, Roy et al. 2023).

[18] Waldrop TG, Eldridge FL, Iwamoto GA, Mitchell JH. Central neural control of respiration and circulation during exercise. Comprehensive Physiology. 2011;333-380.

[19] Roy D, Mazumder O, Jaiswal D, Ghose A, Khandelwal S, Mandana KM, et al. In-silico cardiovascular hemodynamics model to simulate the effect of physical exercise. Biomedical Signal Processing and Control. 2023;80:104364.

We also have added a sentence as one of study limitations to lines 345-349 in the Discussion section.

Reviewer #3:

Thank you for reviewing our manuscript. Your feedback has significantly improved the quality of our work. Please find our detailed responses and corresponding revisions highlighted in pink color in the re-submitted documents.

This work presents a computational model to predict cardiopulmonary variables during controlled breathing and physical exercise. Modelling this is challenging because of the physiological interactions and feedback inherent in this problem. The authors have done a commendable job of integrating voluntary/involuntary respiratory control and cardiopulmonary models.

➔ Thank you for your positive comments.

The complexity of the modelling process makes it difficult to be completely sure of the correctness of the results. While agreement with measured data in Figures 3 and 4 is reassuring I have a few concerns. Can the authors comment on the large tidal volumes (> 3 L) seen in during some time periods (and noted by the in manuscript)? Does it suggest the potential for the model to be numerically unstable, which can be a serious concern for making predictions when measurements are unavailable?

➔ We sincerely apologize for insufficient descriptions on the disagreement of tidal volume in Figs. 3 and 4. This disagreement is not caused by numerical instability. Therefore, it is not a serious concern. We used a closed-loop model proposed by Molkov et al. (2014), which includes a robust algorithm to control respiratory autonomously, similarly to living animals and humans. Even if abnormal respiration is generated, the algorithm can promptly return to a stable respiratory state by using mechanical and chemical feedbacks. Thus, after the initial parameters are input to the model, almost 3 cycles of respiration with a time period of approximately 15 s are needed for a stable respiration in the resting state. We included a process until the stable respiration in the original manuscript; however, the process is not a target that we should compare with experimental data. Therefore, we omitted the process from initial to 20 s in Figs. 1-5 of the revised manuscript for valid comparisons between the model results and experimental data. We have added some sentences to lines 575–579 in the Materials and methods section.

Comparisons are made with the mean of the measured values. How variable are the measurements? It is essential to report some measure of the variability (e.g. standard deviations as error bars) for the data. Does the model have the capability to reproduce measurements for an individual by tuning the parameters?

➔ We modified Figs. 1 and 2 in the revised manuscript (corresponding to Figs. 3 and 4 in the original manuscript, respectively) that includes experimental data with standard deviations as error bars. As for the other question, we can reproduce measurements for an individual by tuning the parameters. For example, we increased the systemic arterial resistance by 37% to reproduce the systolic blood pressure of elderly male; we subsequently performed the same simulation as active short-term exercise (STE) in Fig 1 of the revised manuscript (corresponding to Model A). Simulation results showed that systolic blood pressure became 140 mmHg, which is the same as that obtained from experimental data of elderly male (average age is 67 years old) reported in a study by Ishida (2000). We also decreased a parameter, the vital capacity to reproduce the respiratory state of adult females and then performed the same simulation as active STE in Fig. 1 of the revised manuscript (corresponding to Model B). We have added some sentences to lines 102–116 in the Results section.

In Figure 5 why is there no comparison with experimental measurements of HR and BP from Song et al (2003) and Nuckowska (2019) studies? Also, the HR for the contorl-to-6npm case varies from 100bpm to 70 bpm over the course of less than 5 seconds. Is this realistic? Do any data support this?

➔ The references of Song et al. (2003) and Nuckowska (2019) do not include any time history data. Thus, we have added experimental data from Song et al. (2003), including the peak, mean, and trough values of HR for each controlled BR to Fig 3 (d) of the revised manuscript (corresponding to Fig. 5 of the original manuscript to compare HR variation between model results and their experimental data. As for the other question, the HR varies from 100 bpm to 70 bpm over the course of < 5 s for the control-of-6 bpm as you pointed out. This behavior replicates the cardiac oscillations during deep slow breathing at 6 bpm as reported by Sevoz-Couche and Loborde (2022). We have added some sentences to lines 257–261 in the Discussion section.

The critic-actor reinforcement learning model is not one I am familiar with. It would be useful to provide a brief general description of the ideas behind it. For example, it does not seem use any pre-training. How does that work?

➔ As you have accurately pointed out, we did not use any pre-training. We used the so-called online learning method where the actor alters respiratory muscle activity and then the critic evaluates the results, updating the actor's and critic's weights to increase the reward at each time step. We have added the keyword of online learning to lines 532–533 and 569–570 in the Materials and methods.

In terms of organisation, Figures 1 and 2, associated text and implementation should be in the Materials and Methods. The results would be easier to follow if the experimental design for each of cases 1, 2, 3 were included with the corresponding results section.

➔ We moved Figs. 8 and 9 in the revised manuscript (corresponding to Figs. 1 and 2 in the original manuscript to the Materials and Methods section and added some sentences to lines 79–87 in the Results for case 1, active or passive STE, lines 138–147 in the Results for case 2, VB, and lines 166–175 in the Results for case 3, MS loading.
